# Identification of potential therapeutic target *SPP1* and related RNA regulatory pathway in FECD through bioinformatics

**DOI:** 10.1016/j.isci.2026.115591

**Published:** 2026-04-03

**Authors:** Fuji Deng, Zhixiang Yan, Jinpeng Li, Longwang Wu, Yong Liu, Yuli Yang

**Affiliations:** 1 Southwest Hospital/Southwest Eye Hospital, Third Military Medical University (Army Medical University), Chongqing, China; 2 Chongqing Key Laboratory of Visual Injury and Regeneration, Chongqing, China; 3 School of Computer Science and Technology, Chongqing University, Chongqing, China

**Keywords:** genomics, molecular biology, ophthalmology

## Abstract

Fuchs' endothelial corneal dystrophy (FECD) is a genetic eye disorder that often requires corneal transplantation and lacks targeted therapies. This study aimed to identify new biomarkers and mechanisms of FECD progression. We integrated three GEO corneal endothelial datasets to find consistently differentially expressed genes (DEGs) and constructed a protein-protein interaction (PPI) network to identify hub genes. The identified genes were validated using single-cell RNA sequencing (scRNA-seq) in a rat endothelial injury and endothelial-to-mesenchymal transition (EndMT) model. A competing endogenous RNA (ceRNA) network was predicted using multiple bioinformatics databases. Among 24 common DEGs, Secreted Phosphoprotein 1 (*SPP1*) was identified as a consistently top-ranked upregulated hub gene. This upregulation was validated in an independent dataset (AUC = 0.833) and aligned with the EndMT phase in our model. We propose that *SPP1* is a potential biomarker and therapeutic target for FECD, and the *NEAT1*/miR-181b-5p/*SPP1* axis might be a regulatory RNA pathway involved in FECD development.

## Highlights

• Integrated GEO analysis identified 24 common DEGs and prioritized *SPP1* in FECD
• *SPP1* was an upregulated hub gene with an AUC of 0.833 in the validation dataset
• ScRNA-seq associated *Spp1* upregulation with EndMT in a rat model
• A *NEAT1*/miR-181b-5p/*SPP1* ceRNA axis was predicted in FECD

## Graphical abstract

**Figure:**
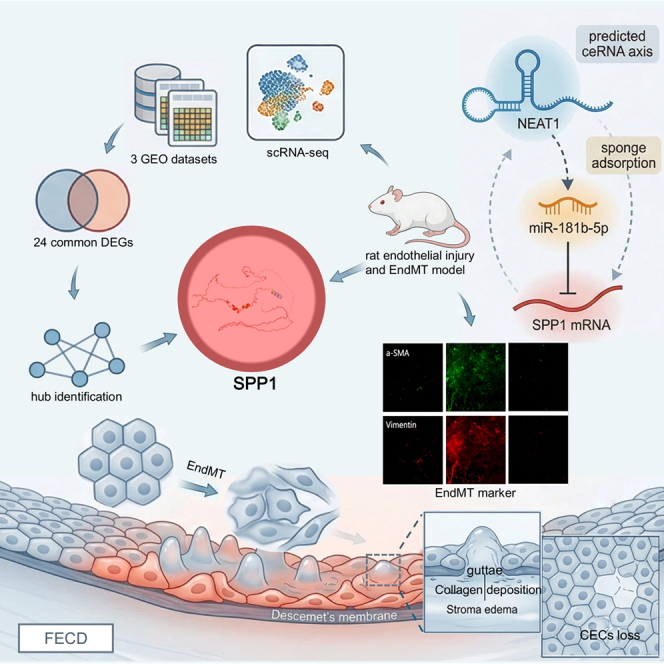

## Introduction

Corneal blindness is the second leading cause of blindness globally, severely impacting the quality of life for tens of millions of people. Among the numerous corneal diseases, Fuchs' endothelial corneal dystrophy (FECD) is a common, age-related, and progressive posterior corneal disorder with a strong hereditary predisposition.^1^ Its core pathophysiology is the functional failure of corneal endothelial cells (CECs). CECs form a non-regenerative monolayer on the posterior surface of the cornea and precisely regulate corneal stromal hydration through their “pump-leak” mechanism, thereby maintaining corneal transparency.^2^

In patients with FECD, CEC density progressively declines, and cells exhibit morphological abnormalities (pleomorphism, polymegathism), accompanied by the formation of characteristic extracellular matrix (ECM) deposits known as guttae on Descemet’s membrane.^3^ The eventual loss of CEC function leads to stromal edema, decreased corneal transparency, and ultimately, severe vision loss.^4^^,^^5^

Etiological studies have revealed a highly complex genetic background for FECD. The abnormal expansion of a CTG trinucleotide repeat in an intron of the *TCF4* gene is the most common and significant genetic risk factor.^6^^,^^7^ Additionally, pathogenic mutations discovered in genes such as *SLC4A11*, *ZEB1*, and *COL8A2* have provided important clues.^8^^,^^9^ However, a critical knowledge gap remains: how are these upstream genetic variations translated into complex downstream cellular pathologies? Specifically, how do they drive CECs to undergo endothelial-to-mesenchymal transition (EndMT), a process where CECs lose their epithelial characteristics and acquire mesenchymal properties, which is widely considered a core mechanism in guttae formation and corneal fibrosis^3^?

Currently, no FDA-approved targeted drug is available globally that can effectively delay or reverse the course of the disease.^4^ Therefore, identifying and validating new therapeutic targets that can intervene in the disease process has become a pressing clinical need. Although some transcriptomic studies have explored FECD, they often suffer from small sample sizes, platform heterogeneity, and a lack of functional validation in specific pathological processes.^10^^,^^11^

To address these limitations, this study employed a multi-layered research strategy. We integrated public data from multiple independent cohorts to screen for robust differentially expressed genes (DEGs). Subsequently, we utilized high-resolution single-cell RNA sequencing (scRNA-seq) in an *in vivo* rat model to link changes in gene expression with the EndMT process. Finally, we explored the upstream non-coding RNA regulatory network. We anticipate that this approach will allow us to prioritize potential hub genes and provide testable mechanistic hypotheses for future therapeutic development.

## Results

**Figure 1:**
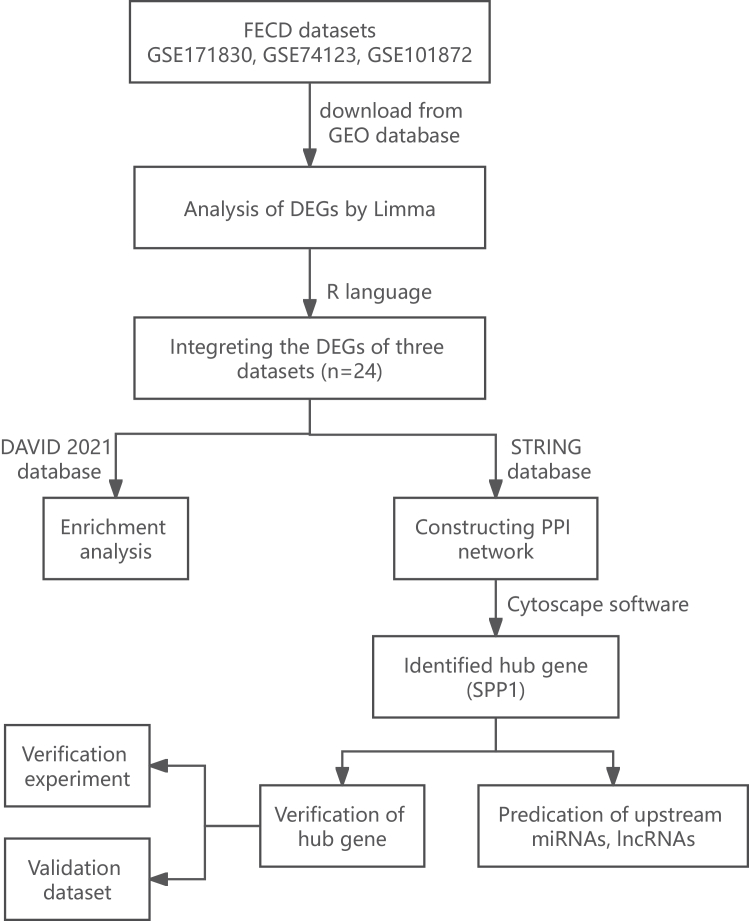
Flow chart of the study screening and selection process Three independent GEO datasets (GSE171830, GSE74123, and GSE101872) were used for integrated DEG screening and PPI network construction, while GSE112039 was used exclusively as an external validation dataset. scRNA-seq was performed on rat corneal endothelial samples at defined post-injury time points to functionally validate hub gene dynamics during EndMT. GEO: Gene expression omnibus; DEGs: differentially expressed genes; *SPP1*: secreted phosphoprotein 1

The flowchart of this study is detailed in Figure 1.

### Screening and identification of DEGs

**Figure 2:**
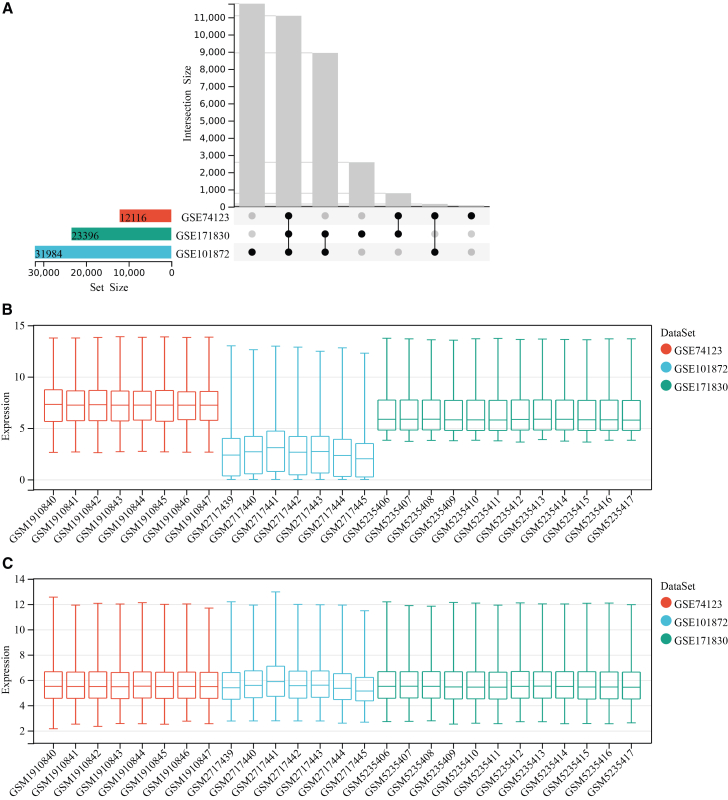
Integration of multiple transcriptomic datasets and correction of batch effects (A) An UpSet plot shows the gene intersections among the three datasets: GSE74123, GSE171830, and GSE101872. (B) Boxplots display the normalized gene expression distributions for all samples before batch correction. The significant variation in the medians and interquartile ranges across these datasets reveals strong batch effects. (C) Corresponding boxplots of gene expression distributions after applying a batch correction algorithm. The resulting alignment of the distributions confirms the effective removal of technical, inter-dataset batch effects, thereby enabling a harmonized analysis. See also Figure S1.

**Figure 3:**
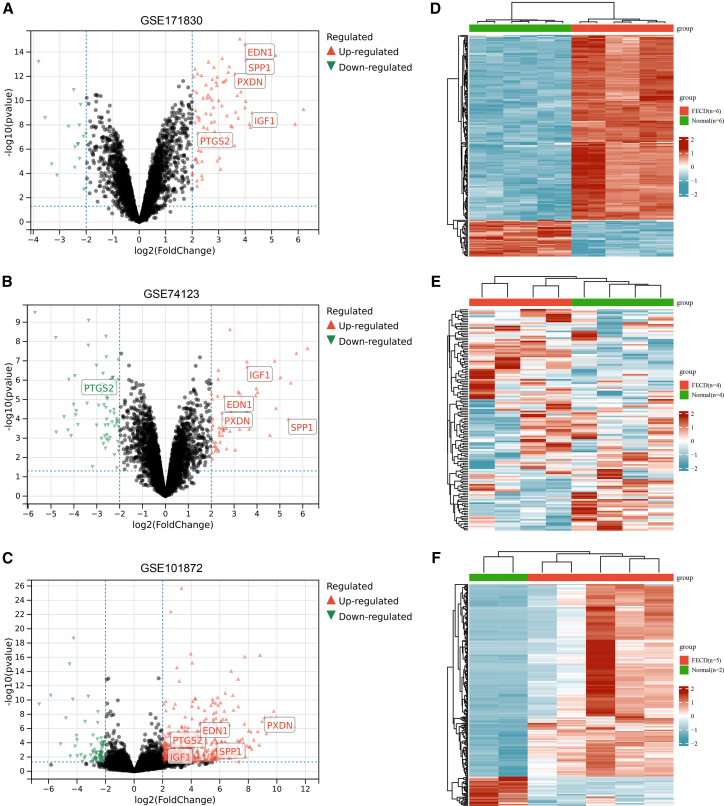
Identification of DEGs in Fuchs' dystrophy versus healthy control DEGs were identified using the criteria of a *p*-value <0.05 and an absolute log_2_(fold change) > 2 (A–C) Volcano plots for each dataset, displaying gene expression magnitude (log_2_(fold change), x axis) versus statistical significance (-log_10_(*p*-value), y axis). Genes meeting these criteria are highlighted, with upregulated genes in red and downregulated genes in blue. (D–F) Heatmaps show the expression profiles of the 250 most significant DEGs (ranked by *p*-value) for each dataset. Each row represents a gene and each column a sample, with color intensity corresponding to row-wise Z-scores of expression (red: high; blue: low).

**Figure 4:**
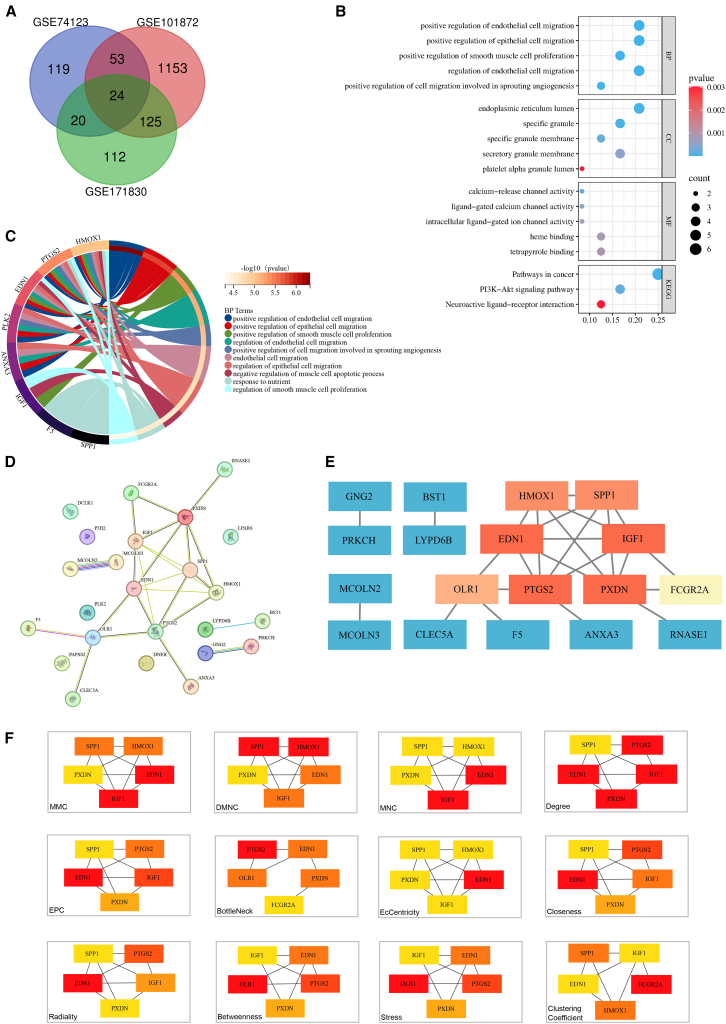
Functional annotation and network analysis of core DEGs (A) A Venn diagram shows the intersection of DEGs from the three datasets (GSE171830, GSE74123, and GSE101872). This intersection identified a core set of 24 DEGs for subsequent analysis. (B) Functional enrichment analysis of the core DEGs, presented as a bubble plot displaying the top enriched GO terms (BP, CC, MF) and KEGG pathways. Dot size reflects the gene count, color indicates the *p*-value, and the x axis represents the gene ratio (the proportion of core DEGs annotated to a term). (C) A chord diagram illustrates the relationships between individual DEGs and the most significantly enriched BP terms. (D) A PPI network of the core DEGs was constructed using the STRING v12.0 with a medium confidence score (>0.4) and visualized in Cytoscape v3.10.3. Nodes represent the DEG-encoded proteins, and edges represent interactions, with edge thickness being proportional to the interaction score. (E–F) Identification of hub genes using 12 topological algorithms in the CytoHubba plugin. The panels highlight the top-ranked genes from various methods (e.g., MCC and degree), identifying them as potential key regulators. GO: Gene ontology; BP: biological process; CC: cellular component; MF: molecular function; KEGG: Kyoto Encyclopedia of Genes and Genomes; PPI: protein-protein interaction. See also Figure S2.

After normalization and differential analysis of the three training datasets (GSE171830, GSE74123, GSE101872), we identified 281, 216, and 1355 DEGs, respectively (Tables 1 and S1, Figures 2, 3, and S1). Through integrated analysis using a Venn diagram, we ultimately identified 24 common DEGs present across all datasets (Figure 4A). These genes were considered to be the core molecules with stable expression changes during FECD pathogenesis.

**Table 1:** Characteristics of the GEO datasets included for analysis

Category	GEO accession	Platform	Specimen	FECD	Normal	DEG	
						Up	Down
Training sets	GSE171830	GPL10558	CE	6	6	235	46
	GSE74123	GPL6244	CE	4	4	112	104
	GSE101872	GPL11154	CE	5	2	1183	172
Validation set	GSE112039	GPL13607	CE	6	6	–	–

GEO: Gene Expression Omnibus; CE: corneal endothelium.

With a more permissive criterion (|log_2_FC| > 1, *p* < 0.05), common DEGs expanded from 24 to 231 in the cross-cohort intersection (Figure S2A, Table S2). Importantly, all 24 core DEGs identified under the stringent threshold were contained within the 231-gene set (24/231, 10.4%).

### Functional and pathway enrichment analysis

GO enrichment analysis revealed that these 24 DEGs were primarily enriched in BP, such as the “positive regulation of endothelial cell migration” and “regulation of smooth muscle cell proliferation.” In terms of CC, they were mainly associated with the “endoplasmic reticulum lumen” and “specific granule membrane.” For MF, they were closely linked to “calcium-release channel activity” and “heme binding” (Table 2, Figures 4B and 4C). KEGG pathway analysis indicated that these DEGs were significantly enriched in key pathways related to cell proliferation and signal transduction, such as “pathways in cancer” and the “PI3K-Akt signaling pathway.”

**Table 2:** Top 5 enriched GO terms and KEGG pathways for core DEGs

Ontology	ID	Description	*p*-Value	Count
BP	GO:0010595	positive regulation of endothelial cell migration	4.63e−7	5
BP	GO:0010634	positive regulation of epithelial cell migration	1.88e−6	5
BP	GO:0048661	positive regulation of smooth muscle cell proliferation	5.03e−6	4
BP	GO:0010594	regulation of endothelial cell migration	7.38e−6	5
BP	GO:0090050	positive regulation of cell migration involved in sprouting angiogenesis	1.02e−5	3
CC	GO:0005788	endoplasmic reticulum lumen	3.24e−5	5
CC	GO:0042581	specific granule	4.01e−5	4
CC	GO:0035579	specific granule membrane	2.00e−4	3
CC	GO:0030667	secretory granule membrane	5.00e−4	4
CC	GO:0031093	platelet alpha granule lumen	3.00e−3	2
MF	GO:0015278	calcium-release channel activity	2.00e−4	2
MF	GO:0099604	ligand-gated calcium channel activity	5.00e−4	2
MF	GO:0005217	intracellular ligand-gated ion channel activity	7.00e−4	2
MF	GO:0020037	heme binding	8.00e−4	3
MF	GO:0046906	tetrapyrrole binding	9.00e−5	3
KEGG	hsa05200	pathways in cancer	2.95e−6	6
KEGG	hsa04151	PI3K-Akt signaling pathway	1.78e−4	4
KEGG	hsa04080	neuroactive ligand-receptor interaction	3.02e−3	3

GO: Gene Ontology; BP: biological process; CC: cellular component; MF: molecular function; KEGG: Kyoto Encyclopedia of Genes and Genomes.

Comparative gene enrichment results revealed that a permissive threshold of an absolute log2(fold change) > 1 introduced more immune-related genes, whereas a stringent threshold selected genes related to collagen formation and cell migration with disease specificity. In 231 DEGs, the top enriched BP terms were dominated by immune activation and proliferation, whereas CC terms highlighted secretory granule membranes and collagen-containing ECM. MF terms included ECM structural constituent, enzyme and peptidase inhibitor activity, and complement binding, and KEGG analysis identified complement and coagulation cascades and phagosome-related pathways (Figure S2B, Table S3).

### PPI network construction and hub gene prioritization

We uploaded the 24 common DEGs to the STRING v12.0 database and constructed a protein-protein interaction (PPI) network comprising 24 nodes and 25 edges (PPI enrichment *p*-value = 1.01e−08) (Figure 4D). Using 12 different algorithms in CytoHubba combined with a weighted consensus ranking approach, five genes (*IGF1*, *EDN1*, *PXDN*, *SPP1*, and *PTGS2*) consistently appeared among the top-ranked candidates (Figures 4E and 4F, Tables 3 and S4). They were thus designated as preselected core hub genes (Table 4).

**Table 3:** Identification and ranking of Hub Genes by 12 CytoHubba algorithms

Algorithm	Gene name
MCC	*IGF1, EDN1,****SPP1****, HMOX1, PXDN*
DMNC	***SPP1****, HMOX1, IGF1, EDN1, PXDN*
MNC	*IGF1, EDN1, PXDN,****SPP1****, PTGS2*
Degree	*PXDN, IGF1, EDN1, PTGS2,****SPP1***
EPC	*EDN1, IGF1, PTGS2,****SPP1****, PXDN*
BottleNeck	*PTGS2, EDN1, OLR1, PXDN, IGF1*
EcCentricity	*EDN1, PXDN, IGF1,****SPP1****, OLR1*
Closeness	*EDN1, PTGS2, IGF1, PXDN,****SPP1***
Radiality	*EDN1, PTGS2, IGF1, PXDN,****SPP1***
Betweenness	*OLR1, PTGS2, EDN1, PXDN, IGF1*
Stress	*OLR1, PTGS2, EDN1, PXDN, IGF1*
Clustering Coefficient	*FCGR2A,****SPP1****, HMOX1, IGF1, EDN1*

The gene ***SPP1*** is highlighted in bold because its consistent high ranking across multiple CytoHubba algorithms.

**Table 4:** Preselected core hub genes

Gene symbol	Full name	GSE101872		GSE171830		GSE74123	
		Log_2_(FC)	*p-*Value	Log_2_(FC)	*p-*Value	Log_2_(FC)	*p-*Value
*IGF1*	insulin-like growth factor I	2.35	7.07e−4	4.26	9.62e−10	3.57	2.00e−7
*EDN1*	big endothelin-1	4.63	1.39e−7	4	2.13e−15	2.57	6.79e−6
*PXDN*	peroxidasin homolog	9.14	2.50e−8	3.61	5.96e−13	2.47	4.76e−5
*SPP1*	secreted phosphoprotein 1	5.80	1.10e−4	4.03	4.07e−14	5.37	1.04e−4
*PTGS2*	prostaglandin G/H synthase 2	2.59	8.14e−4	2.21	4.44e−8	−2.05	9.54e−7

FC: fold change.

Consistent with the stringent screening, PPI analysis of the 231 common DEGs revealed a densely connected core structure. Importantly, all five preselected hub genes (*IGF1*, *EDN1*, *PXDN*, *SPP1*, and *PTGS2*) remained centrally located in the degree-ranked concentric network (Figure S2C). Moreover, *SPP1* remained highly ranked across multiple centrality metrics, including betweenness, bottleneck, and stress (Figure S2D).

### Hub gene validation and diagnostic value assessment

**Figure 5:**
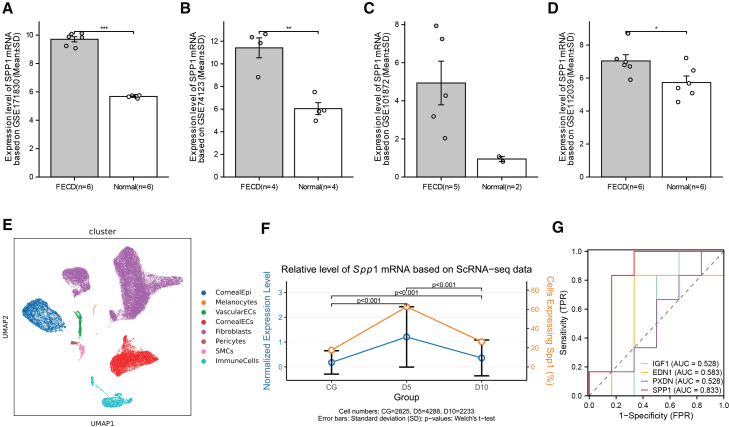
Validation of *SPP1* as a Hub gene and its single-cell characterization in corneal endothelial dysfunction (A–D) Differential expression analysis of *SPP1* between FECD and healthy controls using four independent bulk RNA-seq datasets. (A, B, D) Bar plots demonstrate significantly elevated *SPP1* transcript levels in FECD samples from GSE171830, GSE74123, and the validation cohort GSE112039. Student’s t-tests were utilized for the comparison. (C) A similar increase was observed in GSE101872, but statistical testing was not performed due to the limited control sample size (*n* = 2). Data are shown as mean ± SD. ∗*p* < 0.05, ∗∗*p* < 0.01, and ∗∗∗*p* < 0.001. (E) UMAP visualization of 49,857 cells from a rat corneal injury model analyzed by scRNA-seq. Each color represents a distinct cluster, highlighting the heterogeneity of corneal tissue. (F) Time-course expression of rat *Spp1* in corneal endothelial cells post-injury, based on scRNA-seq data from Days 0, 5, and 10 (pooled from 10 rats per group). (G) ROC analysis of hub genes using *in vitro* cultured cells from the validation dataset GSE112039 (*n* = 6 FECD vs. *n* = 6 controls) identified human *SPP1* as the top predictor in the *in vitro* setting (AUC = 0.833). *PTGS2* was not available in GSE112039 due to probe coverage. See also Figures S3 and S4.

In the independent validation set (GSE112039), *SPP1* mRNA was significantly elevated in cultured FECD samples (*p* = 0.0373) compared to controls (Figure 5D, Table S5), consistent with the training sets (Figures 5A–5C). ROC analysis supported *SPP1* as a promising discriminator (AUC = 0.833) with the highest Youden index, balanced sensitivity, and specificity among these preselected hub genes in this *in vitro* setting (Figures 5G and S3, Table S6).

**Figure 6:**
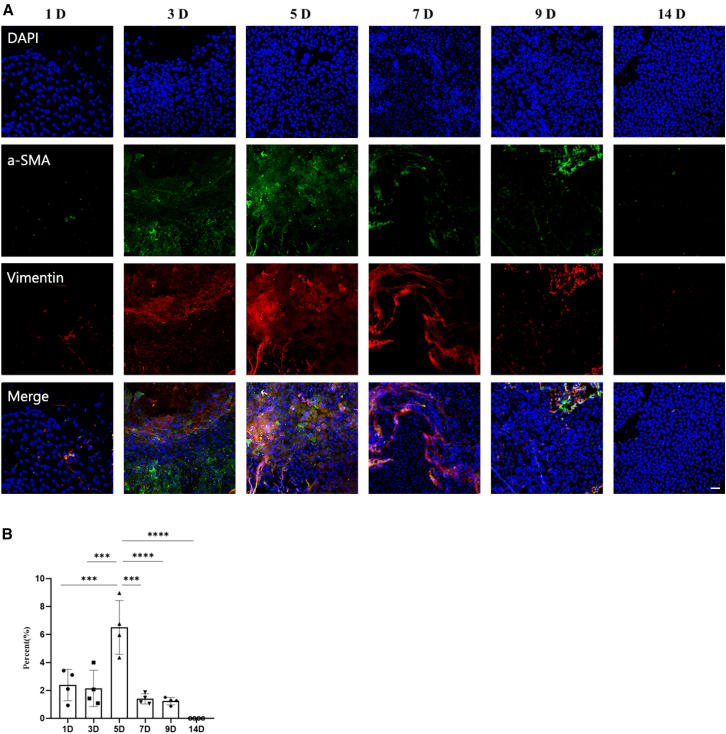
Time course analysis of EndMT marker expression following corneal endothelial injury (A) Representative immunofluorescence images of corneal endothelium at 1, 3, 5, 7, 9, and 14 days after injury (*n* = 10 per group). Tissues were stained for α-SMA (green), Vimentin (red), and nuclei (DAPI; blue). Progressive accumulation and cytoskeletal localization of α-SMA and Vimentin indicate an active EndMT phenotype. Scale bars, 50 μm. (B) Quantification of normalized mean fluorescence intensity for α-SMA and Vimentin across all time points. Data show a significant time-dependent increase in both markers, peaking at day 5 after injury. One-way ANOVA followed by Tukey’s post-hoc test was utilized for the comparison, and data are shown as mean ± SD. ∗*p* < 0.05, ∗∗*p* < 0.01, ∗∗∗*p* < 0.001, and ∗∗∗∗*p* < 0.0001.

Crucially, in our rat corneal injury model, scRNA-seq revealed that *Spp1* expression in the corneal endothelial subpopulation significantly increased at D5 and D10, peaking at D5 (Welch’s *t* test, *p* < 0.05) (Figures 5E, 5F, and S4). This dynamic change was highly consistent with the expression trend of EndMT markers (α-SMA/Vimentin) (Figure 6), suggesting that *Spp1* is closely associated with EndMT-related remodeling in the corneal endothelium.

### In silico prediction of the ceRNA regulatory network

**Figure 7:**
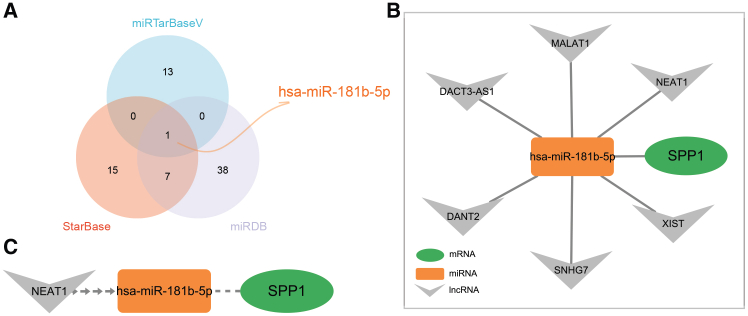
In silico prediction and construction of an *SPP1*-centric ceRNA regulatory network (A) Identification of miRNAs targeting *SPP1*. Putative regulatory miRNAs were predicted using three established online databases: miRTarBase v10.0, StarBase v3.0, and miRDB. The Venn diagram illustrates the overlap among these prediction sets, identifying hsa-miR-181b-5p as the only miRNA common to all three. (B) Construction of the *SPP1*-miRNA-lncRNA ceRNA network. A putative regulatory network was built based on the predicted interaction between *SPP1* and hsa-miR-181b-5p. lncRNAs predicted to act as sponges for hsa-miR-181b-5p were identified via StarBase v3.0 and incorporated into the network. In the visualized network, nodes represent the hub gene (*SPP1*, green ellipse), the key miRNA (hsa-miR-181b-5p, orange rectangle), and candidate lncRNAs (gray diamonds). Edges indicate predicted regulatory interactions, including miRNA targeting or sponging. (C) The potential RNA regulatory pathway *NEAT1*/miR-181b-5p/*SPP1*. Based on the ceRNA network and existing literature supporting the role of *NEAT1* as a molecular sponge, we propose a new regulatory axis. In this hypothesis, the lncRNA *NEAT1* functions as a ceRNA, sequestering hsa-miR-181b-5p and thereby relieving its inhibitory effect on *SPP1* mRNA, ultimately increasing *SPP1* protein expression. miRNA: microRNA; ceRNA: competing endogenous RNA; lncRNA: long non-coding RNA.

Integrated analysis identified hsa-miR-181b-5p as the sole miRNA predicted by all three databases to target *SPP1* (Figure 7A). Further prediction identified lncRNAs, including *NEAT1*, capable of binding hsa-miR-181b-5p. Based on these computational predictions, we constructed a potential *NEAT1*/hsa-miR-181b-5p/*SPP1* competing endogenous RNA (ceRNA) regulatory axis (Figures 7B and 7C).

## Discussion

This study, through a systematic framework integrating large-scale bioinformatics mining and high-resolution single-cell analysis, identifies *SPP1* as a consistent transcriptomic hallmark of FECD and characterizes its dynamic induction during injury-associated EndMT *in vivo*. Our findings suggest that *SPP1* is a key molecule associated with FECD progression and propose a hypothetical upstream regulatory RNA axis for further investigation.

### *SPP1* as a candidate regulator in FECD: Multi-dimensional evidence

We prioritized *SPP1* as a central hub gene based on corroborating evidence. First, its upregulation is robust across three independent patient cohorts and one validation dataset, highlighting it as a reproducible molecular event in FECD. Second, its biological function aligns with FECD pathology. *SPP1* is a matricellular protein known to promote fibrosis in various organs by activating PI3K/Akt and MAPK pathways via integrin binding.^12^^,^^13^ This is consistent with the excessive ECM deposition (guttae) seen in FECD.^14^^,^^15^

Most importantly, our study links *SPP1* to EndMT *in vivo*. While previous studies identified *SPP1* in FECD transcriptomes, we utilized time-resolved scRNA-seq to demonstrate that rat *Spp1* expression peaks specifically during the active phase of EndMT. This suggests that *SPP1* is not merely a passive marker but may be functionally involved in the endothelial dedifferentiation and fibrotic remodeling processes that characterize advanced FECD.

Although other genes such as *IGF1*, *EDN1*, and *PXDN,* were also identified as hubs, *SPP1* was prioritized due to its consistent top-tier ranking across 12 topological algorithms and its distinct expression peak correlating with the phenotypic onset of EndMT in our animal model. However, we acknowledge that FECD likely involves a coordinated network, and these other hubs (representing stress signaling and repair) are likely complementary contributors.

### The *NEAT1*/miR-181b-5p/*SPP1* axis: A predicted hypothesis

To explore the mechanism of *SPP1* upregulation, we constructed a predicted ceRNA network. We propose a model where the lncRNA *NEAT1* sponges hsa-miR-181b-5p, thereby relieving the repression of *SPP1*. *NEAT1* is upregulated in various ocular fibrotic conditions,^16^^,^^17^ and miR-181b-5p is a known regulator of fibrosis that directly targets *SPP1* in other systems, such as aortic valve calcification and airway remodeling.^18^^,^^19^ Furthermore, the downregulation of the miR-181 family has been observed in FECD tissues.^20^

We acknowledge the debate regarding *NEAT1* as a ceRNA, given its predominant nuclear localization. However, studies in other disease contexts suggest that *NEAT1* can influence cytoplasmic mRNA stability through indirect mechanisms or minor cytoplasmic pools under specific stress conditions.^21^ Thus, while this axis remains an in silico-predicted hypothesis in the context of FECD, it provides a biologically plausible framework for future experimental validation (e.g., via dual-luciferase reporter assays and CLIP-seq).

In summary, this study identifies *SPP1* as a robustly upregulated hub gene associated with the EndMT transcriptional program in corneal endothelial injury. We further hypothesize a *NEAT1*/miR-181b-5p/*SPP1* regulatory axis. These findings offer potential molecular targets for therapeutic intervention in FECD, pending further validation in human tissues and chronic disease models.

### Limitations of the study

Several limitations of this study must be acknowledged to contextualize our findings.

First, and most critically, we did not provide protein-level validation (e.g., immunohistochemistry or western blot) of *SPP1* in human FECD surgical specimens. While our bioinformatic results are robust across multiple cohorts, the lack of histological confirmation means that the specific localization of *SPP1* in human corneal endothelium and its spatial relationship with guttae remain to be definitively proven.

Second, the use of a rat mechanical injury model warrants caution. While this model effectively recapitulates acute endothelial injury and EndMT (key components of late-stage FECD), it does not reproduce the chronic, age-dependent, and genetically driven nature of human FECD (e.g., *TCF4* expansion). Therefore, our *in vivo* data support *SPP1*’s role in “endothelial injury and EndMT” rather than the initiation of FECD itself.

Third, the external validation dataset (GSE112039) consisted of *in vitro* cultured cells. Culture conditions are known to alter gene expression profiles, particularly stress and ECM-related genes, which may bias ROC estimates. Thus, these results should be viewed as supportive rather than conclusive evidence of diagnostic performance.

Fourth, the proposed ceRNA network is purely predictive. The functional interaction between *NEAT1*, miR-181b-5p, and *SPP1* has not been experimentally verified in CECs. Future studies utilizing genetic perturbation (knockdown/overexpression) are required to establish causality.

Finally, the heterogeneity of the integrated GEO datasets (varying platforms and patient characteristics) and the small sample sizes of individual cohorts may introduce statistical bias.

## Resource availability

### Lead contact

Requests for further information and resources should be directed to the lead contact, Yuli Yang (yangyuli1023@tmmu.edu.cn).

### Materials availability

This study did not generate new unique reagents.

### Data and code availability

• Data: This paper analyzes existing, publicly available data, accessible at [Database: GSE171830, GSE74123, GSE101872, GSE112039]. scRNA-seq data of rat CECs have been deposited at [Mendeley Data] and are publicly available as of the date of publication at [https://doi.org/10.17632/y7x58f8ww8.1].
• Code: This paper does not report original code.
• Other items: Any additional information required to reanalyze the data reported in this paper is available from the lead contact upon request.

## Acknowledgments

The authors gratefully acknowledge the R programming technical support provided by the School of Computer Science and Technology, 10.13039/501100002369Chongqing University. This work was supported by the 10.13039/100014718National Natural Science Foundation of China (82271055), the 10.13039/501100012166National Key R&D Program of China (2024YFA1108700), the 10.13039/100017501Science-Health Joint Medical Research Project of Chongqing (2022 MSX M051), and the Key Project for the Cultivation of Clinical Innovative Technologies of the 10.13039/501100014869First Affiliated Hospital of Army Medical University (2025CXJS05).

## Author contributions

F.D. and Z.Y. drafted the manuscript, participated in the design of the study, and performed the statistical analysis. J.L. provided technical guidance on R programming and participated in creating the figures and charts. L.W. participated in the animal experiments and performed data curation. Y.L. supervised data curation and provided critical resources. Y.Y. conceived of the study, revised it critically for intellectual content, and gave final approval of the version to be published. All authors agree to be accountable for all aspects of the work.

## Declaration of interests

The authors declare no competing interests.

## STAR★Methods

### Key resources table

**Table:** 

REAGENT or RESOURCE	SOURCE	IDENTIFIER
**Antibodies**		

alpha-Smooth Muscle Actin (D4K9N) Rabbit Monoclonal Antibody	Cell Signaling Technology	Cat# 60839; RRID:AB_3720854
Vimentin (D21H3) Rabbit Monoclonal Antibody	Cell Signaling Technology	Cat# 5741; RRID:AB_10695459
Goat Anti-Rabbit IgG H&L (Alexa Fluor® 488)	Abcam	Cat# ab150077; RRID:AB_2630356
Goat Anti-Rabbit IgG H&L (Alexa Fluor® 568)	Abcam	Cat# ab175695; RRID:AB_2832960

**Critical commercial assays**		

GEXSCOPE® Single Cell Transcriptome Library Preparation Kit	Singleron Biotechnologies	N/A

**Deposited data**		

Training set	https://www.ncbi.nlm.nih.gov/geo/query/acc.cgi?acc=GSE171830	GSE171830
Training set	https://www.ncbi.nlm.nih.gov/geo/query/acc.cgi?acc=GSE74123	GSE74123
Training set	https://www.ncbi.nlm.nih.gov/geo/query/acc.cgi?acc=GSE101872	GSE101872
Validation set	https://www.ncbi.nlm.nih.gov/geo/query/acc.cgi?acc=GSE112039	GSE112039
Raw and analyzed scRNA-seq data of rat	This paper; Mendeley Data	https://doi.org/10.17632/y7x58f8ww8.1

**Oligonucleotides**		

Sprague-Dawley rats	Slaik Jingda Laboratory Animal Co., Ltd	RGD_737903

**Software and algorithms**		

R v4.2.1	https://www.r-project.org/	N/A
Cytoscape v3.10.3	https://cytoscape.org/	N/A
STRING v12.0	https://string-db.org/	N/A
DAVID 2021	https://davidbioinformatics.nih.gov/	N/A
miRTarBase v10.0	https://mirtarbase.cuhk.edu.cn/	N/A
StarBase v3.0	https://rnasysu.com/encori/	N/A
miRDB	https://mirdb.org/	N/A
Sangerbox v3.0	http://sangerbox.com/	N/A

### Experimental model and study participant details

All animal procedures were reviewed and approved by the Laboratory Animal Welfare and Ethics Committee of Army Medical University (approval AMUWEC20224004). Sprague-Dawley rats (180-200g, male) were used for this study at 5 weeks of age. The rats were bred and housed at the Central Laboratory of the Army Medical University facilities on a 12h/12h light-dark cycle with free access to food and water.

### Method details

#### Data Acquisition and DEG filtration

The data were sourced from the Gene Expression Omnibus (GEO).^22^ We employed strict inclusion/exclusion criteria to minimize heterogeneity and ensure data quality. We selected three independent FECD datasets (GSE171830,^23^ GSE74123,^24^ and GSE101872^25^) as the training set based on the following criteria.

1. Specimen Source: Must be human corneal endothelial or Descemet-related specimens.
2. Comparison: Must include both FECD and non-pathologic healthy controls.
3. Data Availability: Must provide a complete expression matrix with gene-symbol annotation suitable for cross-study integration. Datasets lacking controls, using non-human samples, profiling miRNAs only, or without an integrable mRNA matrix were excluded.

This rigorous selection resulted in a total of 15 FECD corneal endothelial samples and 12 healthy control samples for the training set. Additionally, the GSE112039 dataset (cultured FECD vs. control CECs) was selected as an independent validation set to assess gene expression in an *in vitro* model (Tables 1 and S1).

All raw data were normalized. To address batch effects across the heterogeneous cohorts, we used the ComBat algorithm (sva package) with the dataset as the batch variable, while preserving disease status (FECD vs. control) in the model matrix. Batch correction was evaluated by expression distribution and low-dimensional embedding (Figure S1). DEGs were identified using limma v3.40.2 with screening criteria of a *p*-value <0.05 and an absolute log2(Fold Change) > 2. A Venn diagram was used to intersect DEGs to obtain a stable set of common DEGs.^26^

To evaluate the robustness of DEG identification and to minimize the potential loss of moderately but consistently deregulated genes, a supplementary analysis using a more permissive threshold (|log_2_FC| > 1, *p* < 0.05) was performed. The resulting gene lists and downstream network analyses are reported in the Supplementary Materials. (Figure S2, Tables S2 and S3).

#### Functional enrichment analysis

To investigate the biological functions of the DEGs, we used the DAVID 2021 (Database for Annotation, Visualization and Integrated Discovery) database^27^ for Gene Ontology (GO) and Kyoto Encyclopedia of Genes and Genomes (KEGG) pathway enrichment analysis. GO analysis covers three domains: Biological Process (BP), Cellular Component (CC), and Molecular Function (MF). The enrichment results were visualized as bubble plots using the R package ggplot2 v3.3.5 and as chord diagrams using the online platform Sangerbox 3.0 to intuitively display significantly enriched terms.^28^

#### Hub gene identification

We used the STRING v12.0 database to analyze protein interactions.^29^ The minimum required interaction score was set to 0.4. The PPI network data were then imported into Cytoscape v3.10.3^30^ for visualization and analysis. To robustly identify hub genes, we employed 12 different topological algorithms (e.g., MCC, DMNC, Degree) in the CytoHubba plugin.^31^ We selected the top 5 genes ranked by each algorithm and computed a group-weighted consensus ranking to prioritize the most significant hub candidates (Table S4).

#### Animal model and hub gene validation

To investigate the dynamic expression of hub genes during endothelial stress and remodeling, we established a rat corneal endothelial mechanical injury model. This model simulates acute endothelial loss and the subsequent EndMT process, which are relevant to the fibrotic aspects of FECD pathology. Male Sprague-Dawley rats underwent central corneal injury. Corneas were collected at days 1, 3, 5, 7, 9, and 14 for immunofluorescence (α-SMA, Vimentin) to determine key EndMT time points (Day 5 and Day 10). scRNA-seq was performed on pooled corneal endothelium samples (*n* = 10 rats per pool) at CON, D5, and D10. Scanpy v1.8.1 was used for Quality Control (QC) and downstream analyses. Cells with <200 detected genes, cells in the top 2% for gene/UMI counts, and cells with mitochondrial reads >20% were excluded; genes detected in <5 cells were removed. Data were library-size normalized and log-transformed; the top 2,000 highly variable genes were used for PCA (top 23 PCs), followed by Louvain clustering (resolution = 1.2) and UMAP visualization. Batch effects across time points and libraries were corrected using Harmony (v1.0) on the top PCs. After QC, a total of 49,857 high-quality cells were obtained for downstream analysis. CEC subpopulations were identified using a reference-marker strategy (Cell-ID with SynEcoSys marker sets and canonical markers^32^), and the expression of the hub gene *Spp1* at different time points was analyzed.

#### ceRNA regulatory network construction

We used miRTarBase v10.0,^33^ StarBase v3.0,^34^ and miRDB^35^ to predict upstream miRNAs regulating *SPP1*. The prediction results from each database were intersected to identify the most likely targeting miRNA. Subsequently, StarBase v3.0 was used again to predict upstream lncRNAs that interact with this miRNA. The screening criteria included: species Homo sapiens, genome assembly hg38, and a stringency ≥5. Finally, Cytoscape v3.10.3 was used to visualize the predicted “lncRNA-miRNA-mRNA” ternary relationship and construct the ceRNA regulatory network.

### Quantification and statistical analysis

All statistical analyzes were performed using R v4.2.1. Datasets were primarily assessed using limma v3.40.2. For comparisons between two groups, an independent samples *t* test or Welch’s *t* test was used. Receiver Operating Characteristic (ROC) curves and the Area Under the Curve (AUC) were used to evaluate the diagnostic accuracy of the hub gene as an FECD biomarker. We report AUC with 95% confidence intervals estimated by stratified bootstrap resampling (boot. *n* = 5000), and provide the empirical bootstrap AUC distributions (Table S6, Figure S3). One-way ANOVA followed by Tukey’s post-hoc test was used to compare fluorescence intensity. In all tests, a *p* < 0.05 was considered statistically significant (∗*p* < 0.05; ∗∗*p* < 0.01; ∗∗∗*p* < 0.001; ∗∗∗∗*p* < 0.0001).
