# Supplementary material for: Identification of potential therapeutic target SPP1 and related RNA regulatory pathway in FECD through bioinformatics
Source: iScience. 2026 Apr 3;29(5):115591. doi: 10.1016/j.isci.2026.115591 (PMC13099362; doi:10.1016/j.isci.2026.115591)
Supplement: Document S1. Figures S1–S4 and Tables S1–S6 [file mmc1.pdf]

iScience, Volume *29*

Supplemental information

Identification of potential therapeutic target *SPP1* and related RNA regulatory pathway in FECD through bioinformatics

Fuji Deng, Zhixiang Yan, Jinpeng Li, Longwang Wu, Yong Liu, and Yuli Yang

SUPPLEMENTAL FIGURES AND LEGENDS

Figure S1

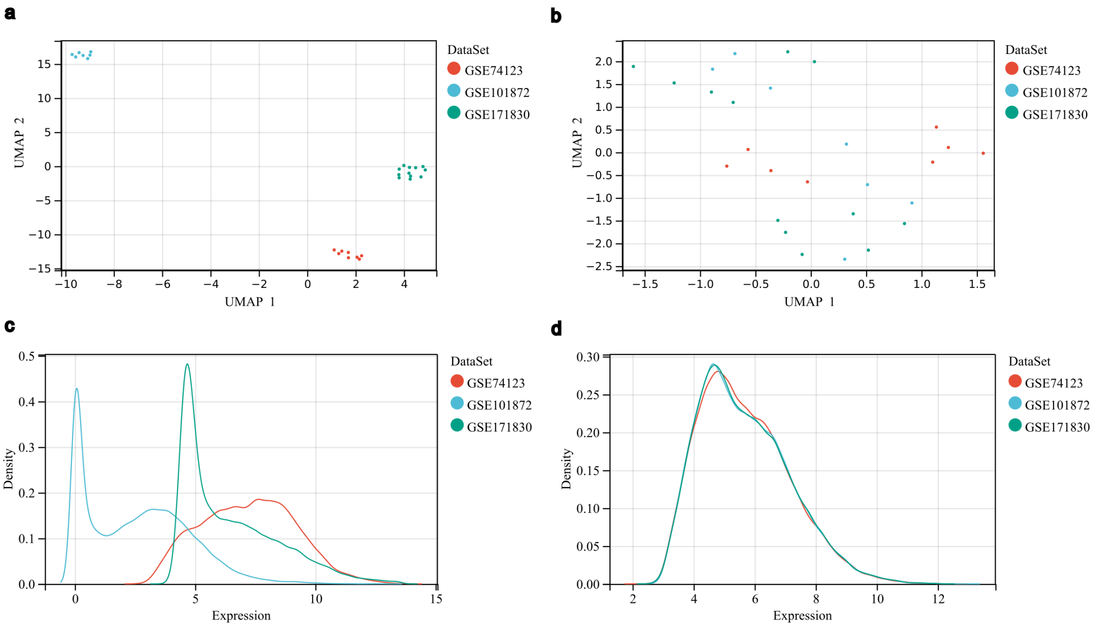

Figure S1. Evaluation of batch effects using UMAP and density plots, related to Figure 2
(a) UMAP plot of sample distributions before batch correction. (b) UMAP plot of sample distributions after batch correction. (c) Density plots before batch correction. (d) Density plots after batch correction.

Figure S2

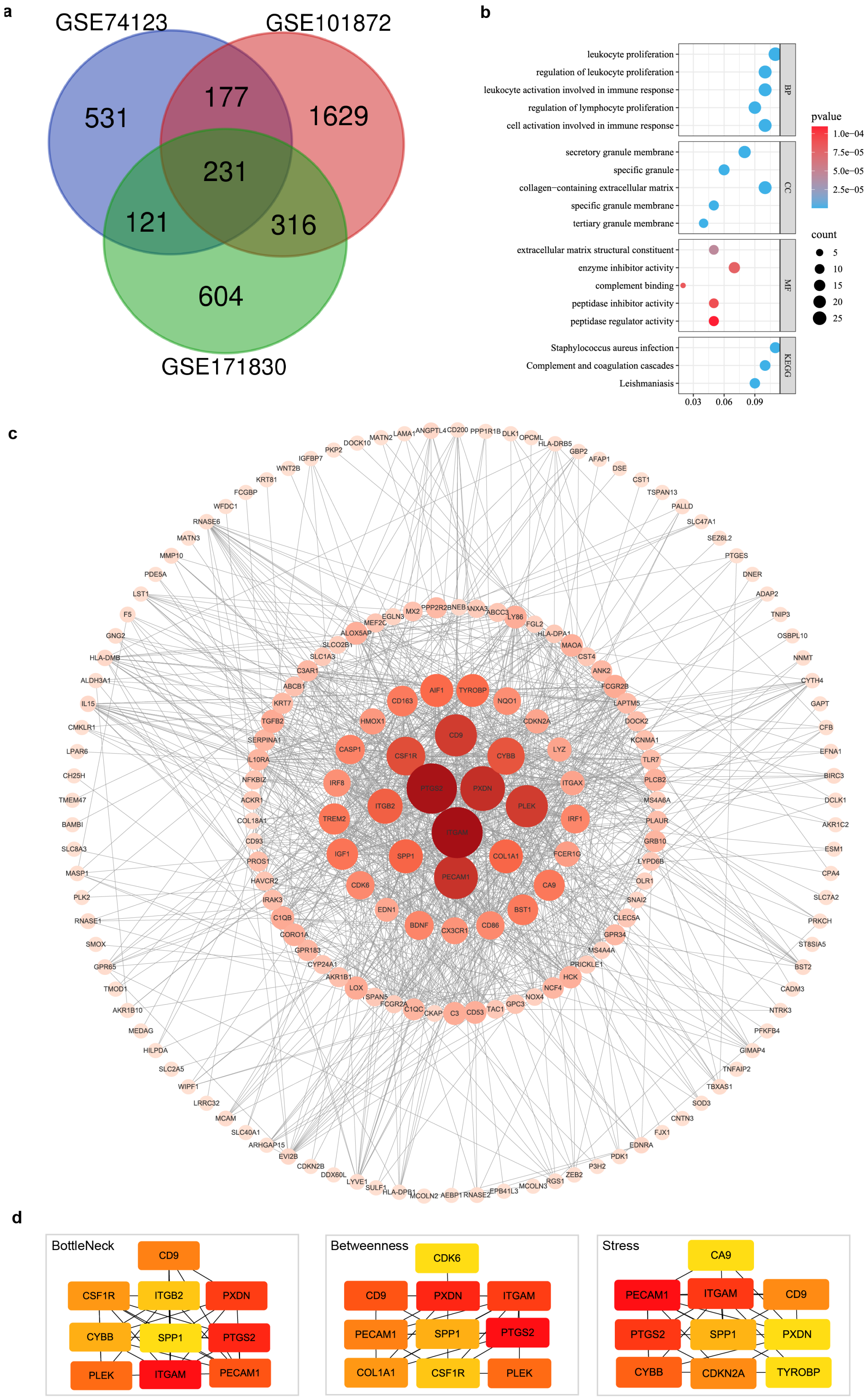

a
GSE74123
GSE101872
GSE171830
531
177
1629
231
121
316
604
b
leukocyte proliferation
regulation of leukocyte proliferation
leukocyte activation involved in immune response
regulation of lymphocyte proliferation
cell activation involved in immune response
secretory granule membrane
specific granule
collagen−containing extracellular matrix
specific granule membrane
tertiary granule membrane
extracellular matrix structural constituent
enzyme inhibitor activity
complement binding
peptidase inhibitor activity
peptidase regulator activity
Staphylococcus aureus infection
Complement and coagulation cascades
Leishmaniasis
BP
CC
MF
KEGG
0.03
0.06
0.09
pvalue
1.0e−04
7.5e−05
5.0e−05
2.5e−05
count
5
10
15
20
25
c
AIF1
TYROBP
CD163
NQO1
HMOX1
CD9
CDKN2A
CASP1
CSF1R
CYBB
LYZ
IRF8
PTGS2
PXDN
ITGAX
ITGB2
PLEK
TREM2
IRF1
ITGAM
IGF1
SPP1
COL1A1
FCER1G
PECAM1
CDK6
CA9
EDN1
BST1
BDNF
CX3CR1
CD86
d
BottleNeck
CD9
CSF1R
ITGB2
PXDN
CYBB
SPP1
PTGS2
PLEK
ITGAM
PECAM1
Betweenness
CDK6
CD9
PXDN
ITGAM
PECAM1
SPP1
PTGS2
COL1A1
CSF1R
PLEK
Stress
CA9
PECAM1
ITGAM
CD9
PTGS2
SPP1
PXDN
CYBB
CDKN2A
TYROBP

Figure S2. Hub DEGs identification with a permissive threshold ($|\log_2 FC| > 1$, $p < 0.05$), related to Figure 4

(a) Venn diagram shows the expanded list of 231 DEGs. (b) The enriched GO chord plot of the CC, MF and KEGG signaling pathways of DEGs. The findings highlight immune-related and extracellular matrix remodeling pathways. (c) Degree-ranked concentric network was constructed with the DEGs in Cytoscape v3.10.3. Preselected hub genes (*IGF1*, *EDN1*, *PXDN*, *SPP1*, and *PTGS2*) remained centrally positioned. (d) Betweenness, BottleNeck, and Stress were used to analyze the top 10 genes with high scores. *SPP1* retained high rankings.

Figure S3

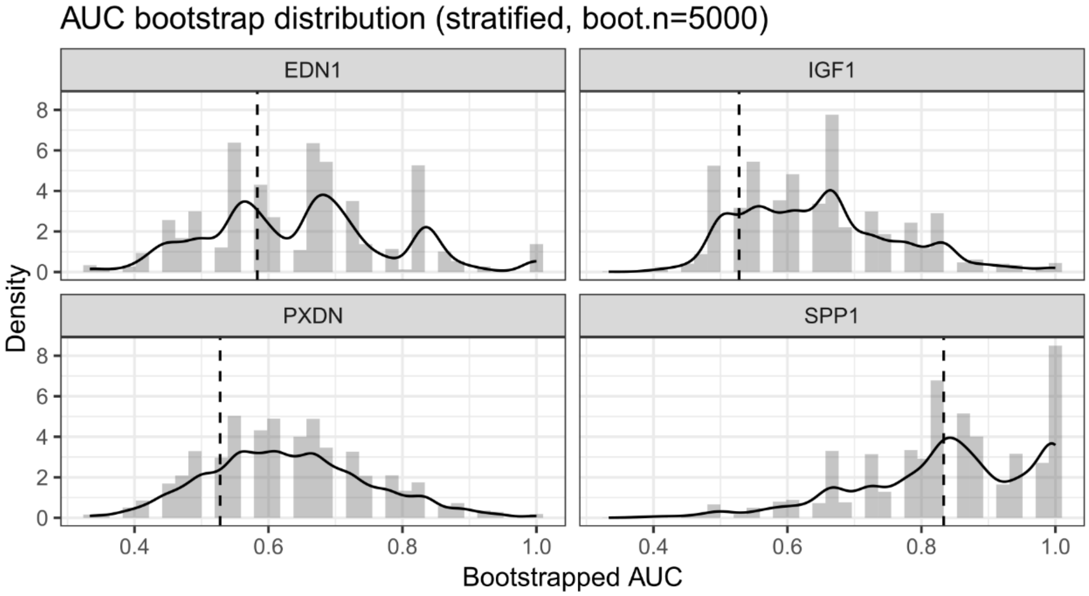

Figure S3. Stratified bootstrap distribution of AUC values, related to Figure 5g

Figure S4

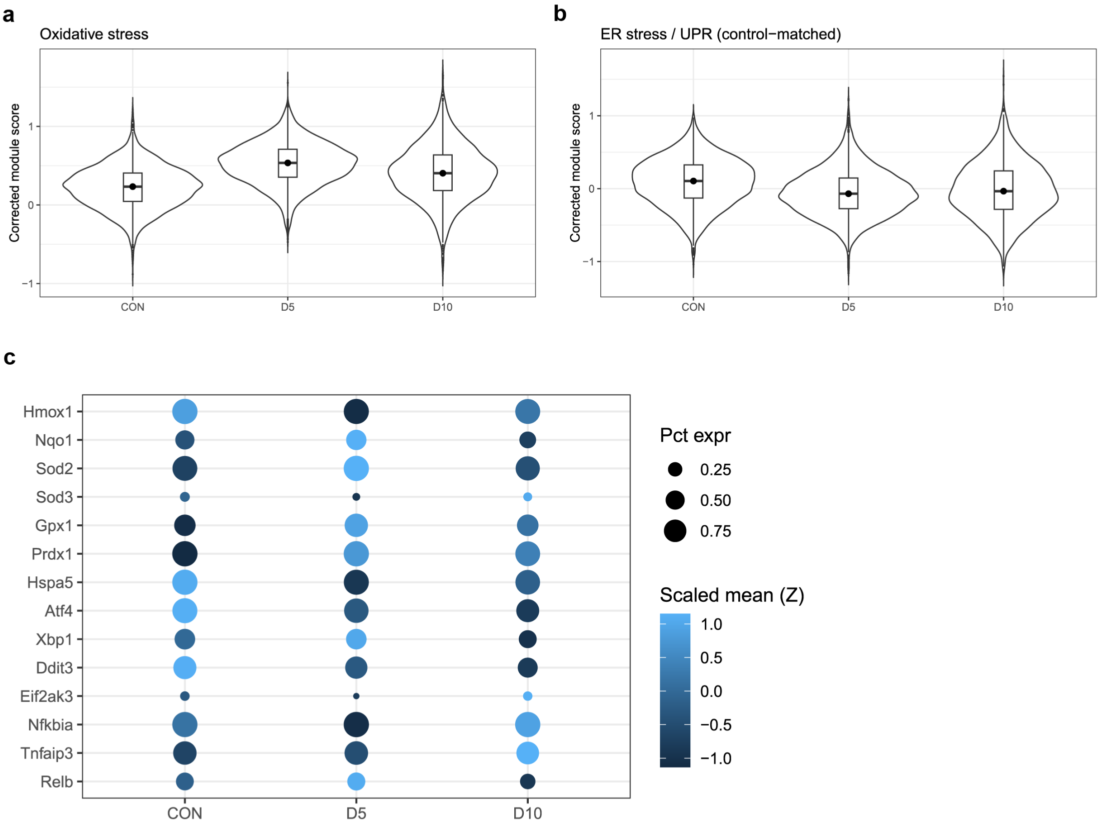

Figure S4. Time-course changes in oxidative stress and ER stress/UPR module activity in the rat endothelium from sc-RNA seq data, related to Figure 5e

(a) Corrected oxidative stress module scores. (b) Corrected ER stress/UPR module scores. control-matched per-cell gene-set scoring. Module scores were calculated via single-cell gene set enrichment analysis (scGSEA). (c) Dot plot showing expression patterns of key genes involved in oxidative stress and ER stress/UPR across time points.

SUPPLEMENTAL TABLES

Table S1. Human transcriptomic datasets summary, related to Table 1

GEO dataset	Sample size (FECD vs Control)	Demographics (age, sex)	FECD stage	Genotype info
GSE171830	Arrays：<br>3 pooled FECD vs 3 control， duplicate → 6 FECD vs 6 control arrays;<br><br>Biological specimens：<br>9 FECD（3 pools × 3 each）vs 3 Control	FECD: 65.1 ± 5.5;<br>Female: Male 6:3.<br>Controls: 79.3 ± 6.7;<br>Female: Male 1:2	Grades 3－6<br>(modified Krachmer scale)	Not reported
GSE74123	n=4 FECD vs n=4 Control	FECD: 75.5 ± 4.0;<br>Female: Male 3:1.<br>Control: 52.8 ± 18.8;<br>Female: Male 0:4	Stage II－IV<br>(Symptomatic late-onset FECD)	Not reported
GSE101872	n=5 FECD vs n=2 Control	Not reported	Grades 0－6<br>(modified Krachmer scale)	FECD with TCF4 repeat expansion
GSE112039	n=6 FECD vs n=6 Control	FECD: 68.8±9.9;<br>Female: Male 2:4.<br>Control: 68.7 ± 9.5;<br>Female: Male 4:2	Not reported	Not reported

Sample information of GSE112039 was extracted from GEO annotations without linked primary publication.

Table S2. List of DEGs, related to Figure 4

$\lvert Log_2(FC) \rvert > 2$, $p < 0.05$ (n=24)	$\lvert Log_2(FC) \rvert > 1$, $p < 0.05$ (n=231)
EDN1, IGF1, PAPSS2, ANXA3, PXDN, MCOLN2, BST1, SPP1, PTGS2, OLR1, GNG2, LYPD6B, HMOX1, FCGR2A, P3H2, MCOLN3, RNASE1, F5, CLEC5A, PRKCH, DCLK1, PLK2, LPAR6, DNER	*KCNA1, TNIP3, EDN1, NCF4, IGF1, SLC2A5, HLA-DRB5, RPS6KA5, PAPSS2, SLCO2B1, PALLD, ANXA3, COL1A1, TSPAN13, MAOA, C3, PXDN, EDNRA, BAMBI, PTGES, CD53, FCGBP, MCOLN2, ARHGAP15, BST1, HCK, OSBPL10, PPP1R1B, CAPN6, VIT, GLDN, DSE, NFKBIZ, CASP1, SPP1, CA9, PTGS2, OLR1, GLIPR2, CHN2, CD200, CD93, TAC1, LST1, CMKLR1, CPA4, GNG2, ITGB2, EPB41L3, CDKN2B, PROS1, VEGFA, LAMA1, CD86, PKP2, NNMT, SAMD5, RGS1, TP53I3, CYTH4, LYPD6B, CST4, HMOX1, FCGR2A, LRRC32, ANGPTL4, ALOX5AP, TREM2, MS4A4A, AFAP1, LYVE1, TLR7, GIMAP4, P3H2, MCOLN3, TENM3, OPCML, HLA-DPB1, EVI2B, PDE5A, NCKAP1L, AKR1B1, CYBB, FER, ITGAX, KRT7, IL10RA, RNASE1, ADAP2, SLC1A3, LOX, SULF1, LYZ, F5, CLEC5A, NREP, PRKCH, GPC3, HILPDA, MASP1, WIPF1, FCER1G, PFKFB4, DLK1, CX3CR1, ERRFI1, WFDC1, GALNT1, PEAR1, MMP10, HAVCR2, PLAUR, CH25H, MEDAG, MEF2C, ESM1, ST8SIA5, FGL2, TGFB2, C1QC, PLCB2, DOCK2, IRF1, PLEK, RNASE6, AKR1B10, FCGR2B, BDNF, SOD3, DDAH1, SLC8A3, SLC40A1, C3AR1, DCLK1, RNASE2, LY86, IGFBP7, SLC47A1, CST1, SERPINA1, AKR1C2, ABCB1, MS4A6A, CDK6, TMEM47, HLA-DPA1, EFNA1, ANK2, SYT15, RAB23, SMOX, ABCC3, HLA-DMB, KYNU, TBXAS1, MATN3, SEZ6L2, IRAK3, GBP2, NOX4, IL15, MCAM, CD9, NTRK3, DCDC2, AIF1, TSPAN5, KRT81, NEB, GAPT, MX2, CCDC68, SLC7A2, TNFAIP1, ITGAM, CNTN3, CD163, GRB10, KCNT2, TYROBP, GPR65, ECM2, PECAM1, NCKAP5, PLK2, BST2, GPR183, CADM3, ACKR1, ANOS1, NQO1, ALDH3A1, DOCK10, RNF144B, PAMR1, GPR34, CORO1A, AEBP1, SHROOM3, WNT2B, MRGPRX3, ZP4, C1QB, ZEB2, LPAR6, MATN2, TMOD1, ST6GALNAC2, PDK1, DDX60L, MEG3, TNFAIP2, PPP2R2B, LAPTM5, CFB, PCP4, RNF125, EGLN3, IRF8, PRICKLE1, BCAT1, CDKN2A, DNER, MAMDC2, CSF1R, KDM3A, SNAI2, FJX1, CYP24A1, CLIC6, BIRC3*

Table S3. Top 5 Enriched GO Terms and KEGG Pathways for 231 DEGs, related to Table 2

Ontology	ID	Description	*p*-Value	Count
BP	GO:0070661	leukocyte proliferation	1.84e-13	25
BP	GO:0070663	regulation of leukocyte proliferation	3.8e-13	22
BP	GO:0002366	leukocyte activation involved in immune response	3.85e-12	22
BP	GO:0050670	regulation of lymphocyte proliferation	4.35e-12	20
BP	GO:0002263	cell activation involved in immune response	5.09e-12	22
CC	GO:0030667	secretory granule membrane	5.52e-09	19
CC	GO:0042581	specific granule	6.43e-09	14
CC	GO:0062023	collagen-containing extracellular matrix	7.86e-09	22
CC	GO:0035579	specific granule membrane	9.53e-09	11
CC	GO:0070821	tertiary granule membrane	1.83e-07	9
MF	GO:0005201	extracellular matrix structural constituent	4.47e-05	10
MF	GO:0004857	enzyme inhibitor activity	7.82e-05	15
MF	GO:0001848	complement binding	8.42e-05	4
MF	GO:0030414	peptidase inhibitor activity	9.01e-05	10
MF	GO:0061134	peptidase regulator activity	1.10e-4	11
KEGG	hsa05150	Staphylococcus aureus infection	2.92e-10	14
KEGG	hsa04610	Complement and coagulation cascades	7.10e-10	13
KEGG	hsa05140	Leishmaniasis	2.66e-09	12

GO: Gene Ontology; BP: biological process; CC: cellular component; MF: molecular function; KEGG: Kyoto Encyclopedia of Genes and Genomes.

Table S4. Group-weighted consensus ranking of hub genes across 12 CytoHubba algorithms, related to Table 3

Gene	n/12 (Top5)	Local density mean rank (MCC/DMNC/MNC)	Degree/neighborhood mean rank (Degree/EPC/BottleNeck)	Path/distance mean rank (EcCentricity/Closeness/Radiality /Betweenness/Stress)	Clustering coefficient rank	Group-weighted mean rank
EDN1	12	2.67	2	1.8	5	2.87
IGF1	12	1.67	3	3.8	4	3.12
SPP1	9	2.67	5	5.2	2	3.72
PTGS2	8	5.67	2.67	2.8	6	4.28
PXDN	11	4.33	3.33	3.6	6	4.32
FCGR2A	1	6	6	6	1	4.75
HMOX1	3	4	6	6	3	4.75
OLR1	4	6	5	3.8	6	5.2

Table S5. Multiple-testing correction of preselected core hub genes in GSE112039, related to Figure 5d

Gene	Normal mean ± SD (n=6)	FECD mean ± SD (n=6)	$Log_2(FC)$	*p*-Value	*q*-Value
IGF1	4.208±0.723	4.013 ± 0.241	-0.195	0.5539	0.7295
EDN1	4.624 ± 0.564	4.511 ± 0.471	-0.113	0.7138	0.7295
PXDN	8.316 ± 1.367	8.056 ± 1.157	-0.26	0.7295	0.7295
SPP1	5.729 ± 0.968	7.039 ± 0.921	1.31	**0.0373**	0.1493

p-values: Welch's two-sided t-test; *q*-values: BH FDR correction; FC: fold change.

Table S6. Diagnostic performance of hub genes in GSE112039 (ROC-derived metrics), related to Figure 5g

Gene	Cut-off	Sensitivity	Specificity	Youden index	AUC (95% CI)
IGF1	4.1333	0.8333	0.5	0.3333	0.528 (0.134–0.922)
EDN1	4.7747	0.8333	0.6667	0.5	0.583 (0.192–0.974)
PXDN	9.2821	0.8333	0.3333	0.1667	0.528 (0.162–0.894)
SPP1	6.5831	0.8333	0.8333	0.6667	0.833 (0.554–1.000)
